# Construction and validation of nomogram prediction model for recurrent spontaneous abortion based on the expression of MALAT1, miR-515-5p, and MCL1 mRNA

**DOI:** 10.3389/fmed.2025.1558818

**Published:** 2025-12-17

**Authors:** Xufei Chen, Xiaotong Wang, Yuyi Yuan, Shuzhen He, Huanhuan Huang, Chunhua Zhong

**Affiliations:** Department of Obstetrics, The Affiliated Dongguan Songshan Lake Central Hospital, Guangdong Medical University, Dongguan, China

**Keywords:** MALAT1, MCL1 mRNA, miR-515-5p, nomogram prediction model, recurrent spontaneous abortion

## Abstract

**Objective:**

To investigate the construction and clinical application value of Nomogram predictive model for recurrent spontaneous abortion (RSA) based on the expressions of MALAT1 (Metastasis Associated Lung Adenocarcinoma Transcript 1), miR-515-5p, and MCL1 (Myeloid Cell Leukemia-1) mRNA.

**Methods:**

According to the 7:3 ratio, the patients were divided into the training set (*n* = 135) and the validation set (*n* = 58) by the complete random method. In the training set, multivariate Logistic regression was used to analyze the risk factors for RSA and a Nomogram prediction model was constructed based on the expressions of MALAT1, miR-515-5p, and MCL1 mRNA. The receiver operating characteristic (ROC) curve and calibration curve were drawn to evaluate the prediction performance of the Nomogram model and verified in the validation set. At the same time, decision curve analysis (DCA) was used to evaluate the clinical application value of the Nomogram model.

**Results:**

There were 38 cases (28.15%) of RSA in the training set and 17 cases (29.31%) in the validation set. During the training set, advanced maternal age, number of abortions >3, low progesterone level in early pregnancy, high expression of MALAT1, low expression of miR-515-5p and low expression of MCL1 mRNA were the independent risk factors for RSA (*p* < 0.05). We will further construct the Nomogram prediction model. In the training set and validation set, the C-index values of Nomogram model were 0.915 and 0.869, respectively. The calibration curve showed that the predicted values accorded well with the actual values, with the average absolute errors of 0.012 and 0.034, respectively. The *p-*values of Hosmer–Lemeshow test were 0.362 and 0.779, respectively, indicating that the model had good calibration and fitting performance. The ROC curve showed that the Nomogram models in the training and validation sets predicted AUC values for RSA to be 0.916 (95% CI, 0.858–0.974) and 0.867 (95% CI, 0.742–0.992), respectively.

**Conclusion:**

The Nomogram prediction model constructed based on the expressions of MALAT1, miR-515-5p, and MCL1 mRNA is helpful to predict the occurrence of RSA in the early stage and guide the making of appropriate clinical decisions.

## 1 Introduction

Recurrent spontaneous abortion (RSA) refers to the spontaneous abortion that occurs two or more times in a row, and is one of the common complications of pregnancy in clinic. It has caused heavy psychological burden and economic pressure to patients and their families (1). The etiology of RSA is complex, involving genetic factors, endocrine factors, immune factors, infectious factors and anatomical factors, etc. However, the exact mechanism is not completely clear. Therefore, early prediction of RSA is of great significance for formulating effective prevention measures and improving the success rate of pregnancy (2). In recent years, with the rapid development of molecular biology technology, more and more studies have begun to pay attention to the role of non-coding RNA and mRNA in the occurrence of RSA. MALAT1 is a long-chain non-coding RNA (lncRNA) that plays a key role in the progression and origin of a variety of tumors and diseases. Studies have shown that the abnormal expression of MALAT1 is closely related to the occurrence of RSA (3). MiR-515-5p is RNA (microRNA that participates in various biological processes by regulating the expression of target genes). Studies have found that the abnormal expression of miR-515-5p is also associated with the occurrence of RSA (4). Myeloid leukemia 1 (MCL1) encoded by MCL1 mRNA is an anti-apoptotic protein that plays an important role in maintaining cell survival and proliferation. Studies have shown that the abnormal expression of MCL1 is closely related to the occurrence of RSA (5). Notably, bioinformatics predictions using TargetScan 8.0 and StarBase 3.0 tools indicate that MCL1 mRNA is a potential target gene of miR-515-5p, with conserved binding sites present in its 3′-untranslated region (3′-UTR). Meanwhile, MALAT1, as a classical long non-coding RNA, can function as a competing endogenous RNA (ceRNA) by binding and sequestering miRNAs, thereby regulating the expression of downstream target genes (6). Based on this, we hypothesize that MALAT1 may sponge miR-515-5p, attenuating its inhibitory effect on MCL1 mRNA, and consequently maintaining normal levels of MCL1 protein—a key anti-apoptotic factor in trophoblasts. Dysregulation of this ‘MALAT1/miR-515-5p/MCL1 mRNA’ axis (e.g., high MALAT1 expression, low miR-515-5p expression, low MCL1 mRNA expression) may disrupt trophoblast survival and placental development, ultimately contributing to RSA. This integrated regulatory relationship forms the core rationale for our selection of these three molecules to construct the predictive model. Nomogram is a visual graphical tool based on multivariate statistical model to predict the probability of individual clinical practice, which has been widely used in the prediction and evaluation of tumors, cardiovascular diseases and other diseases. The purpose of this study was to explore the construction and clinical application value of an Nomogram prediction model for the occurrence of RSA based on the expressions of MALAT1, miR-515-5p, and MCL1 mRNA, in the hope of opening up a new perspective and strategy for early recognition, prevention and control of RSA.

## 2 Objects and methods

### 2.1 Subjects

A total of 193 patients who received the related examinations of recurrent abortion in our hospital from January 2020 to December 2023 were selected as the research subjects. Inclusion criteria: (1) age 18–45 years old; (2) Two or more consecutive spontaneous abortions; (3) Sign informed consent form and voluntarily participate in this study. (4) All participants were in the non-pregnant state and had no history of miscarriage within 3 months before enrollment; blood samples were collected during pre-pregnancy examination. Exclusion criteria: (1) Genetic diseases or chromosome abnormalities; (2) Severe heart, liver, kidney and other organ diseases; (3) Patients with immune system disease or autoimmune disease; (4) Infections with infectious diseases or active infections; (5) Abnormal anatomical structure or genital tract deformity; (6) Other treatments that may affect the pregnancy outcome are being received. This study has been approved by the Ethics Committee of our hospital.

### 2.2 Main reagents and instruments

Trizol reagent (Invitrogen, the US); Reverse transcription kit (Takara, Japan); SYBR Green PCR Master Mix(Roche, Switzerland); MALAT1, MIRI-515-5P, and MCL1 mRNA primers (Shanghai Shenggong Biotechnology Co., Ltd.); Real-time fluorescent quantitative PCR instrument (ABI7500, the US). The sequences and related information of primers for MALAT1, miR-515-5p, MCL1 mRNA, and internal reference genes (GAPDH/U6) are shown in Supplementary Table 1.

### 2.3 Specimen collection and processing

Five milliliters of fasting peripheral venous blood of the research subjects was drawn, placed in an EDTA anticoagulation tube, mixed upside down gently, centrifuged at 3,000 r/min for 10 min at 4 °C, and the plasma was separated and transferred to a centrifuge tube free of RNA enzyme for storage at −80 °C.

### 2.4 RNA extraction and reverse transcription

Plasma total RNA was extracted according to the Trizol reagent instruction, and RNA concentration and purity were determined by ultraviolet spectrophotometer. RNA samples with A260/A280 ratio of 1.8–2.0 were used for subsequent experiments” is now updated to: “RNA quality was assessed using an Agilent 2100 Bioanalyzer (Agilent Technologies, USA), and only samples with RNA Integrity Number (RIN) > 7.0 and A260/A280 ratio of 1.8–2.0 were used for subsequent experiments. According to the instructions of reverse transcription kit, the extracted total RNA was reversely transcribed into cDNA, and the reaction conditions were as follows: 37 °C for 15 min, 85 °C for 5 s and 4 °C for storage. For miR-515-5p reverse transcription, a stem-loop specific primer was used instead of a random primer to improve detection specificity.

### 2.5 Real-time fluorescent quantitative PCR detection

Before formal experiments, standard curves were constructed using serial dilutions of cDNA to verify amplification efficiency. The amplification efficiencies of MALAT1, miR-515-5p, MCL1 mRNA, GAPDH, and U6 were 98.2, 95.7, 96.5, 99.1, and 97.3%, respectively, all within the range of 90–110%, and the correlation coefficients (*R*^2^) were all >0.99. The SYBR Green fluorescent quantitative PCR method was used to detect the mRNA expression levels of MALAT1, miR-515-5p and MCL1. GAPDH was taken as the internal reference gene, while miR-515-5p took U6 as the internal reference gene. The reaction system consisted of 20 μL, including 10 μL of Sybrgreen PCR Master Mix, 0.8 μL of upstream and downstream primers, 2 μL of cDNA template and 6.4 μL of ddH₂O. The reaction conditions were as follows: pre-denaturation at 95 °C for 5 min; The samples were denatured at 95 °C for 10 s, annealed at 60 °C for 30 s, and extended at 72 °C for 30 s, for a total of 40 cycles. Three multiple wells were set for each sample, and the experiment was repeated three times. The relative mRNA expression levels of MALAT1, miR-515-5p, and MCL1 mRNA calculated using the 2-ΔΔ Ct method.

### 2.6 Data collection

Clinical information such as the age of the patient, pre-pregnancy body mass index (BMI), number of pregnancies, delivery times, number of abortions, gestational week of abortion, type of previous abortion (spontaneous abortion or induced abortion), progesterone level in early pregnancy, chorionic gonadotropin (hCG) level in early pregnancy, and whether or not uterine fibroids and endometriosis were combined were collected.

### 2.7 Statistical methods

SPSS 26.0 and R 4.0.3 were used for data analysis. For continuous variables conforming to normal distribution, we presented them in the form of mean standard deviation (mean SD), and used independent sample t test to compare and analyze between the two groups. Categorical variables were described as frequency or proportion, and chi-square test (*χ*^2^) was used to compare between the two groups. In the aspect of risk factor selection, univariate and multivariate Logistic regression analysis was used to explore the relevant factors for the occurrence of RSA, and accordingly, the Nomogram prediction model was constructed. This model was established using the “rms” package of R language. The “pROC” package was used to draw the receiver operating characteristic (ROC) curve to evaluate the prediction performance of the model, and a consistency index (C-index) was calculated to quantify the accuracy of the model prediction. The model was internally verified, and the Bootstrap resampling method was adopted, and the calibration curve was drawn to visually evaluate the calibration performance of the model. In statistical analysis, *p* < 0.05 were considered statistically significant.

## 3 Results

### 3.1 Comparison of baseline data between training set and validation set

There was no significant difference in baseline data between the training and validation sets (*p* > 0.05) (Table 1).

**Table 1:** Comparison of baseline data between training set and validation set.

Index		Training set (*n* = 135)	Validation set (*n* = 58)	*t/χ^2^*	*p*
Age (years)		27.89 ± 3.24	28.36 ± 3.41	0.909	0.364
Pre-pregnancy BMI (kg/m^2^)		23.16 ± 2.34	23.02 ± 2.03	0.367	0.713
Abortion gestation week		9.24 ± 1.55	9.09 ± 1.60	0.610	0.542
Number of abortions	2–3 times	85 (62.96)	32 (55.17)	1.031	0.309
	>3 times	50 (37.04)	26 (44.83)		
Smoking history	Yes	12 (8.89)	5 (8.62)	0.003	0.952
	No	123 (91.11)	53 (91.38)		
Drinking history	Yes	8 (5.93)	3 (5.17)	0.017	0.895
	No	127 (94.07)	55 (94.83)		
Type of previous abortion	Spontaneous abortion	102 (75.56)	40 (68.97)	0.906	0.341
	Induced abortion	33 (24.44)	18 (31.03)		
Number of pregnancies (times)		3.26 ± 1.21	3.41 ± 1.32	0.768	0.443
Number of deliveries (times)		0.89 ± 0.57	0.78 ± 0.63	1.190	0.235
Combined uterine myoma	Yes	15 (11.11)	8 (13.79)	0.278	0.598
	No	120 (88.89)	50 (86.21)		
Combined hypertension	Yes	7 (5.19)	2 (3.45)	0.023	0.878
	No	128 (94.18)	56 (96.55)		
Combined diabetes	Yes	6 (4.44)	3 (5.17)	0.023	0.878
	No	129 (95.56)	55 (40.74)		
Progesterone in early pregnancy (ng/mL)		20.36 ± 1.82	20.06 ± 1.68	1.073	0.284
HCG in early pregnancy (mIU/mL)		13232.33 ± 446.31	13251.14 ± 443.64	0.268	0.788
MALAT1		1.85 ± 0.57	1.79 ± 0.63	0.649	0.516
miR-515-5p		0.67 ± 0.26	0.65 ± 0.24	0.501	0.616
MCL1 mRNA		1.55 ± 0.43	1.57 ± 0.53	0.275	0.783

All continuous variables are presented as mean ± standard deviation; categorical variables are presented as frequency (percentage).

### 3.2 Analysis of independent risk factors for RSA in training set

Single factor Logistic regression analysis showed that age, number of abortions, progesterone in early pregnancy, MALAT1 expression level, miR-515-5p expression level and MCL1 mRNA expression level had a correlation with RSA (*p* < 0.05), as shown in Table 2. With the occurrence of RSA as the dependent variable (No = 0, Yes = 1), the variables that were statistically significant in the univariate analysis were included in the multivariate Logistic regression analysis. The variable assignment table was shown in Table 3, and the results showed that advanced maternal age, the number of abortions >3, low progesterone level in early pregnancy, high expression of MALAT1, low expression of miR-515-5p, and low expression of MCL1 mRNA were the independent risk factors for the occurrence of RSA (*p* < 0.05), as shown in Table 4.

**Table 2:** Univariate analysis of RSA in the training set.

Index		RSA group (*n* = 38)	Non-RSA (*n* = 97)	*t/χ^2^*	*p*
Age (years)		31.16 ± 1.23	29.51 ± 1.54	2.569	0.011
Pre-pregnancy BMI (kg/m^2^)		23.64 ± 2.13	22.89 ± 2.03	1.904	0.059
Abortion gestation week		8.31 ± 2.12	9.05 ± 2.34	1.695	0.092
Number of abortions	2–3 times	16 (42.11)	65 (67.01)	7.056	0.007
	>3 times	22 (57.89)	32 (32.99)		
Smoking history	Yes	5 (13.16)	7 (7.22)	0.569	0.450
	No	33 (86.84)	90 (92.78)		
Drinking history	Yes	4 (10.53)	6 (6.19)	0.250	0.616
	No	34 (89.47)	91 (93.81)		
Type of previous abortion	Spontaneous abortion	30 (78.95)	79 (81.44)	0.109	0.740
	Induced abortion	8 (21.05)	18 (18.56)		
Number of pregnancies (times)		2.88 ± 1.46	2.54 ± 1.12	1.451	0.149
Number of deliveries (times)		0.64 ± 0.41	0.55 ± 0.38	1.210	0.228
Combined uterine myoma	Yes	11 (28.95)	15 (15.46)	3.192	0.074
	No	27 (71.05)	82 (84.54)		
Combined hypertension	Yes	6 (15.79)	7 (7.22)	1.426	0.232
	No	32 (84.21)	90 (92.78)		
Combined diabetes	Yes	4 (10.53)	3 (3.09)	1.743	0.186
	No	34 (89.47)	94 (96.91)		
Progesterone in early pregnancy (ng/mL)		16.23 ± 3.89	20.21 ± 4.54	4.050	0.001
HCG in early pregnancy (mIU/mL)		13567.12 ± 447.37	13676.37 ± 468.42	1.233	0.219
MALAT1		1.89 ± 0.46	1.68 ± 0.32	3.011	0.003
miR-515-5p		0.38 ± 0.11	0.45 ± 0.15	2.612	0.010
MCL1 mRNA		1.12 ± 0.26	1.30 ± 0.33	3.013	0.003

**Table 3:** Variable assignment table.

Variable	Meaning	Evaluation
X1	Age	Continuous variable
X2	Number of abortions	2–3 times = 0, >3 times = 1
X3	Progesterone in early pregnancy	Continuous variable
X4	MALAT1	Continuous variable
X5	miR-515-5p	Continuous variable
X6	MCL1 mRNA	Continuous variable
Y	RSA occurs	No = 0, Yes = 1

**Table 4:** Multivariate logistic regression analysis of RSA in training set.

Project	*B*	Standard error	*Wald*	*p*	OR	95% CI
Age	0.841	0.177	22.563	0.001	2.319	1.639–3.282
Number of abortions	1.027	0.393	6.824	0.009	2.793	1.292–6.036
Progesterone in early pregnancy	−0.220	0.054	16.511	0.001	0.803	0.722–0.893
MALAT1	1.470	0.543	7.320	0.007	4.350	1.500–12.619
miR-515-5p	−3.838	1.502	6.527	0.011	0.022	0.001–0.409
MCL1 mRNA	−1.943	0.658	8.729	0.003	0.143	0.039–0.520

### 3.3 Establishment of nomogram prediction model

**Figure 1:**
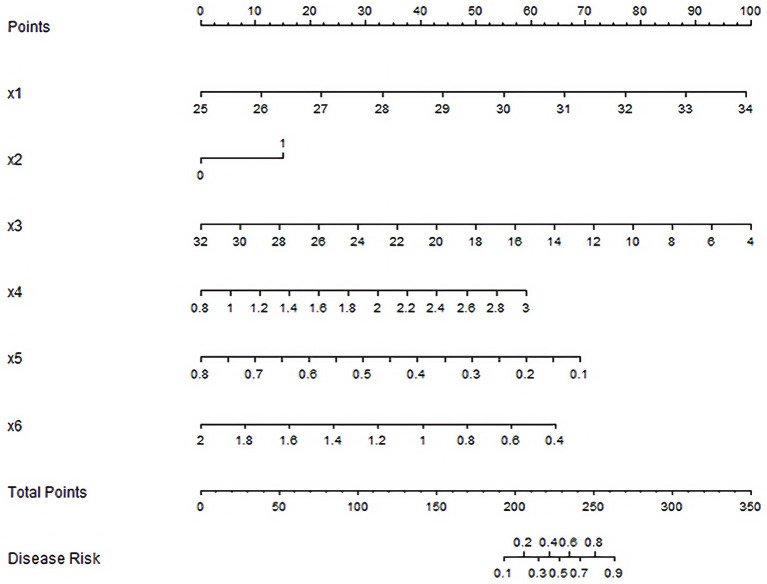
Nomogram prediction model for RSA.

According to the results of multivariate Logistic regression analysis, the Nomogram prediction model was constructed (for the expression levels of MALAT1, miR-515-5p, and MCL1 mRNA, the average expression level of the three genes in plasma samples from non-RSA patients in the training set-calibrated by internal reference genes GAPDH/U6-was set as the reference value of 1), as shown in Figure 1. According to the assignment of each risk factor, the total score was calculated to predict the probability of occurrence of RSA.

### 3.4 Evaluation and validation of nomogram prediction model

**Figure 2:**
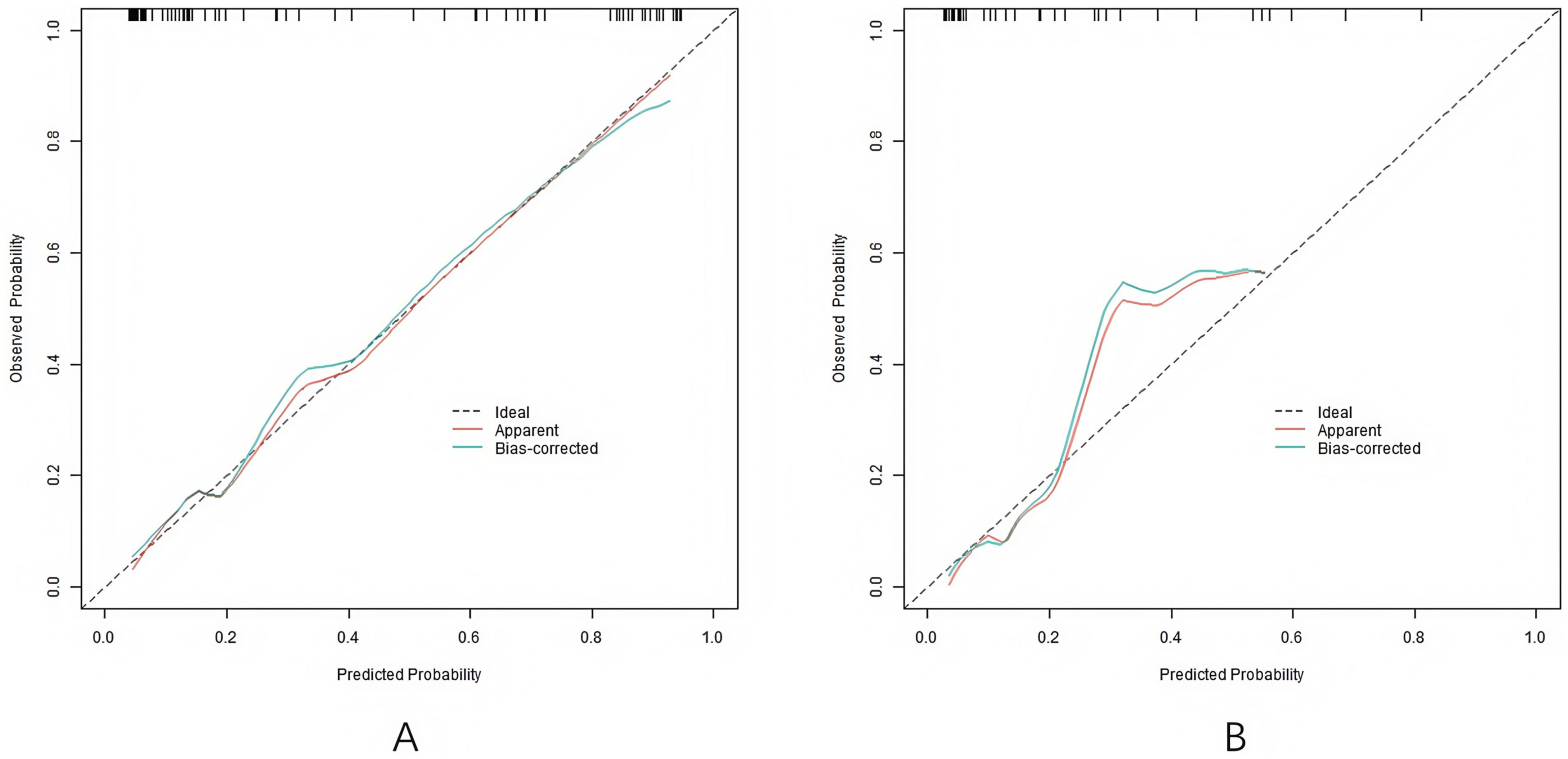
Calibration curves (**A** is the training set, and **B** is the validation set).

**Figure 3:**
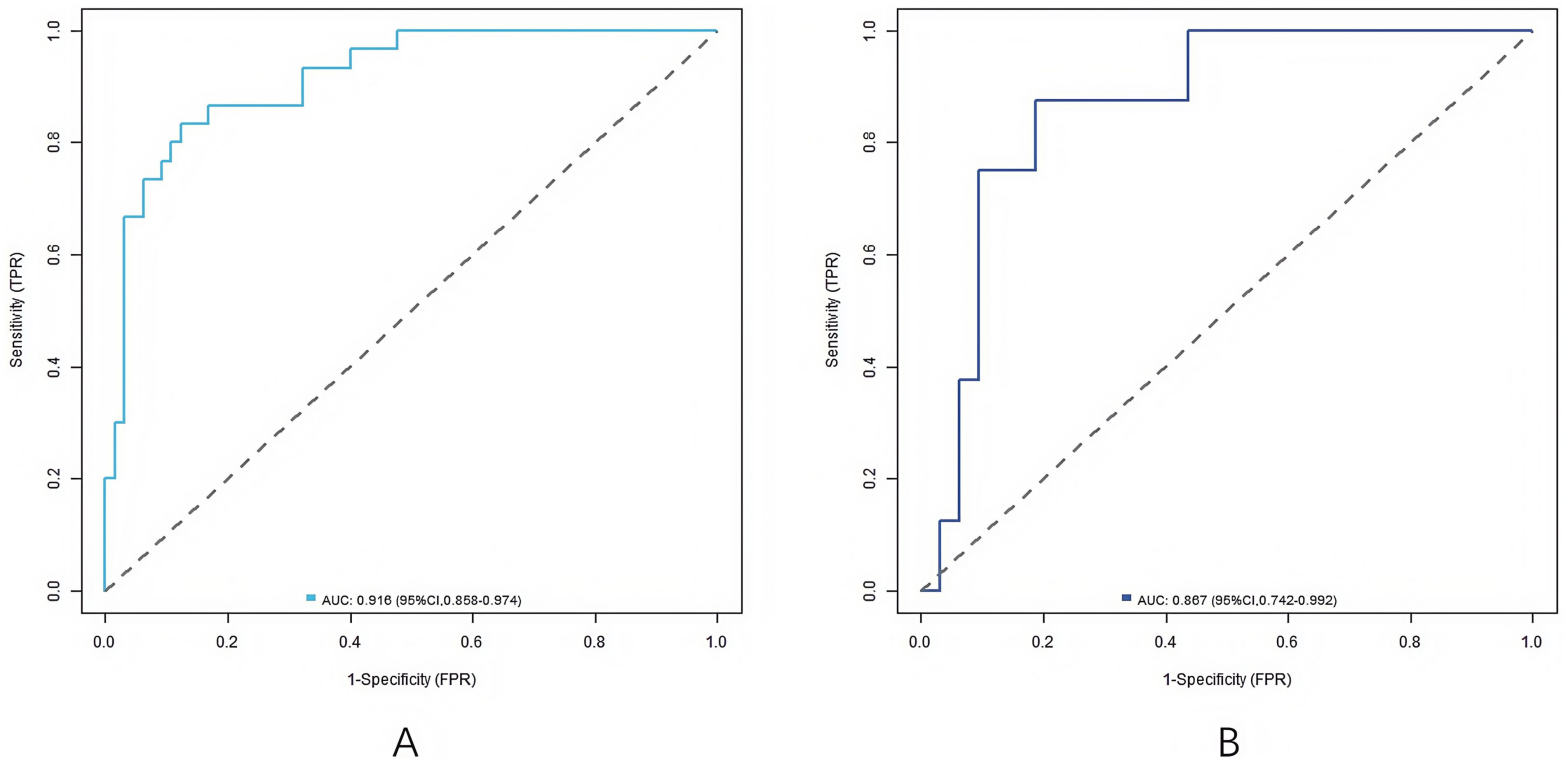
ROC curves (**A** is the training set, and **B** is the validation set).

In the training set and validation set, the C-index values of Nomogram model were 0.915 and 0.869, respectively. The calibration curve showed that the predicted values accorded well with the actual values, with the average absolute errors of 0.012 and 0.034, respectively. The *p* values of Hosmer–Lemeshow test were 0.362 and 0.779, respectively, indicating that the model had good calibration and fitting performance. The ROC curve showed that the AUC of RSA predicted by the Nomogram model in the training set and the validation set were 0.916 (95% CI: 0.858–0.974) and 0.867 (95% CI: 0.742–0.992), respectively, and the sensitivities were 0.833 (training set) and 0.750 (validation set), and the specificities were 0.877 (training set) and 0.875 (validation set), respectively, indicating that the model had high prediction performance, as shown in Figures 2, 3.

### 3.5 Analysis of decision-making curve of nomogram column line prediction model

**Figure 4:**
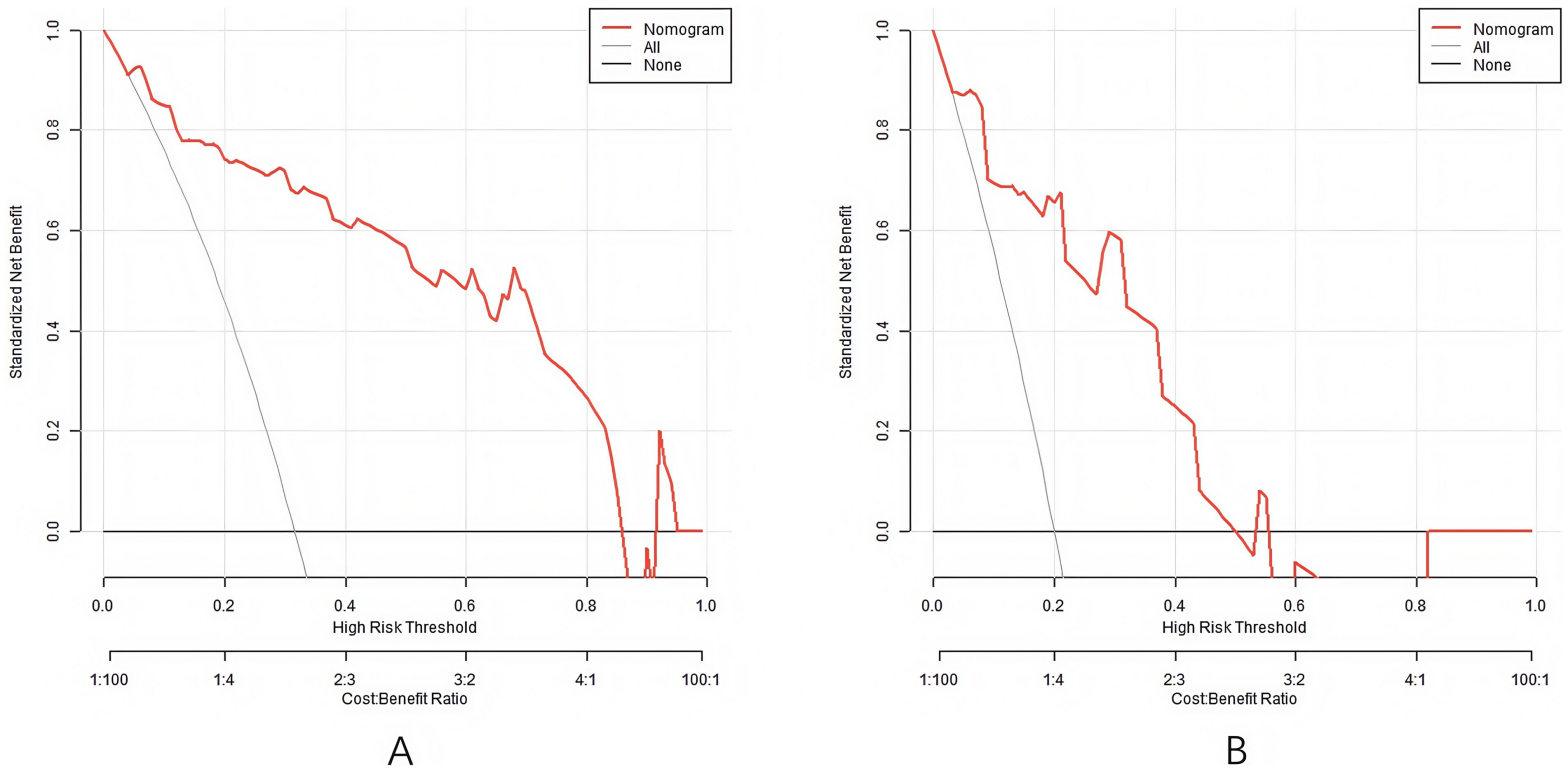
Decision curves (**A** is the training set, and **B** is the validation set).

The decision curve shows that when the threshold is within the range of 0.1–0.9, the application of Nomogram column line model to predict the decisions that occur with RSA has more clinical benefit than the decisions that both occur with or without RSA (Figure 4).

## 4 Discussion

RSA is a thorny problem in clinical practice of obstetrics and gynecology. It not only brings physical injury to patients, but also causes huge psychological burden. Although a large number of studies have been devoted to explore the etiology of RSA, there are still a considerable number of cases with unknown etiology, which makes clinical diagnosis and intervention face many challenges. Therefore, finding biomarkers that can accurately predict the occurrence of RSA and an effective prediction model are crucial for improving the reproductive health outcomes of patients. In this study, we attempted to construct a Nomogram model to predict the occurrence of RSA based on molecular biological markers, which has important clinical significance. MALAT1, miR-515-5p, and MCL1 mRNA, as research subjects, may play key roles in the reproductive process. Through the study of these molecules, we are expected to gain an in-depth understanding of the pathogenesis of RSA and provide the basis for early diagnosis and intervention in clinical practice.

In this study, we found that advanced maternal age, number of abortions, low progesterone in early pregnancy, high expression of MALAT1, high expression of miR-515-5p and low expression of MCL1 mRNA were the independent risk factors for recurrent abortion. The reason is that with the gradual growth of age, the reserve capacity of female ovary shows a downward trend, resulting in the prolonged retention time of eggs in the ovary. In this process, the eggs are interfered with more by the external environment factors, thus increasing the risk of meiosis abnormalities (such as division is not separated). This abnormal division further increases the probability of egg chromosome aneuploidy and negatively affects the quality of germ cells (7). When an egg with abnormal chromosomes is successfully fertilized, the formed embryo often shows low development potential and is prone to spontaneous abortion in the early pregnancy. In addition, the endometrial receptivity (ability to accept embryos) decreases with age. Endometrium plays a central role in the successful implantation of an embryo. Its structural integrity (such as an appropriate thickness), adequate blood perfusion, and appropriate cytokine secretion and other physiological conditions have a vital impact on the implantation process (8). For older women, hormonal-regulated periodic changes in the endometrium may be disrupted, affecting the normal implantation and subsequent development of the embryo, leading to recurrent abortion. Multiple abortions can cause different degrees of damage to the uterus (9). For example, curettage may cause damage to the basal layer of the endometrium, leading to adverse consequences such as decreased endometrial thickness and intrauterine adhesions, which can reduce the available effective surface area for embryo implantation and have a negative impact on embryo implantation. At the same time, repeated uterine operations may also cause cervical dysfunction, in the second and third trimesters of pregnancy unable to bear the weight of the fetus and placenta, prone to abortion. Repeated abortion may break the original endocrine and immune balance in women. In terms of endocrine, it may lead to dysfunction of hypothalamic–pituitary-ovarian axis regulation and affect the normal secretion of sex hormones, for example, corpus luteum insufficiency causes insufficient secretion of progesterone to maintain a normal pregnancy (10). In terms of immunity, it may cause the maternal immune system to abnormally recognize the embryo and produce autoantibodies such as antiphospholipid antibodies, which interfere with placental angiogenesis and blood supply of the embryo and cause abortion. Progesterone is essential to maintain the secretory state of endometrium. In early pregnancy, low level of progesterone cannot fully convert endometrium into an environment suitable for embryo implantation and growth, resulting in unstable embryo implantation and easy shedding from endometrium, triggering abortion (11). Progesterone physiologically plays a key role in inhibiting the contraction of uterine smooth muscle. When the progesterone content in the body is insufficient, the excitability of uterine smooth muscle will be relatively increased, which may lead to the appearance of irregular contraction. Such abnormal contractile activity may exert a pressing effect on the embryo and interfere with its normal blood supply, thus negatively affecting the development of the embryo, and even leading to abortion when severe (12). The ‘MALAT1/miR-515-5p/MCL1 mRNA’ regulatory axis may be a key pathway in RSA pathogenesis. High expression of MALAT1 (as a ceRNA) could sequester miR-515-5p, but in our study, low miR-515-5p expression was observed in RSA patients—this may be due to additional regulatory factors (e.g., other miRNAs or transcription factors) that further reduce miR-515-5p levels. Meanwhile, high MALAT1 may directly interfere with trophoblast migration/invasion (6) and disrupt maternal immune tolerance to the embryo (13), as MALAT1 is involved in immune regulation and its overexpression may break the immune tolerance balance. Reduced miR-515-5p fails to effectively inhibit MCL1 mRNA, but our results showed low MCL1 mRNA in RSA patients, suggesting that MALAT1 overexpression may not fully compensate for MCL1 mRNA downregulation (possibly caused by other mechanisms like promoter methylation). Additionally, low miR-515-5p may disrupt the normal expression of genes related to embryonic development and interfere with embryonic cell differentiation, proliferation, and apoptosis by dysregulating specific cellular signaling pathways necessary for embryonic development (14, 15), further increasing RSA risk. Ultimately, low MCL1 mRNA leads to decreased MCL1 protein, which breaks the dynamic balance of trophoblast apoptosis and proliferation (5), causes increased trophoblast apoptosis, impairs placental development, and fails to provide adequate nutrition and oxygen for the embryo, leading to embryonic developmental disorders and abortion (16).

Nomogram predictive model constructed based on multiple risk factors can comprehensively evaluate the multi-dimensional influencing factors of RSA, and provides an intuitive and quantitative prediction aid for clinicians. In the training set and the validation set, the C-index index of the model reached 0.915 and 0.869, respectively, showing a good discrimination, which indicated that the model had high prediction accuracy and could effectively distinguish patients with RSA from non-RSA patients. The results of calibration curve analysis showed that the predicted values and the actual values had a high degree of agreement, and the average absolute errors of the training and validation set were 0.012 and 0.034, respectively, further verifying the accuracy and reliability of the model. *p*-values of Hosmer–Lemeshow test in the training and validation set were 0.362 and 0.779, respectively, indicating that the model had good goodness of fit and there was no significant difference between the predicted results and the actual observed values. In addition, ROC curve analysis showed that the area under the curve (AUC) of the training and validation set were 0.916 (95% CI: 0.858–0.974) and 0.867 (95% CI: 0.742–0.992), respectively. The sensitivity and specificity of the model were satisfactory, and the AUC value was close to 1, demonstrating the high prediction performance of the model. Decision curve analysis (DCA) further confirms the value of the model in clinical application, and can provide a powerful reference for clinical decisions and assist doctors to assess the potential benefits of preventive interventions for high-risk patients with RSA. However, this study also has certain limitations. The limited number of samples from a single medical institution may lead to selection bias and limit the general applicability of the study results. Future studies need to expand the sample size and conduct multi-center studies to include patient groups from different regions and ethnic groups to further verify and optimize the prediction model (17). In addition, although multiple influencing factors were considered, there may be confounding factors that were not found or included in the analysis, such as environmental factors and other potential molecular markers, which may affect the results of the study. Further exploration is required in the future (18). This study has certain limitations that should be considered. First, the specific action mechanisms of MALAT1, miR-515-5p, and MCL1 mRNA in the occurrence and development of RSA have not been explored in depth. Although our study confirms a correlation and proposes an integrated hypothesis, the molecular mechanism of their interaction and how they cause abortion by regulating cell function and signaling pathways remain unclear. In the future, through in-depth research on cell experiments and animal models, we can provide a more comprehensive theoretical basis for the pathogenesis of RSA (19). Furthermore, it is important to note that this is an observational study. The identified associations between the biomarkers and RSA do not equate to causal relationships. Further functional experiments (e.g., utilizing trophoblast cell models or RSA animal models) are necessary to verify whether the dysregulation of these molecules directly causes RSA. Finally, regarding the clinical applicability of our model, the single-center design and the moderate sample size may limit the generalizability of our findings and pose a risk of overfitting for a multivariate model with six predictors. Although the model demonstrated good performance in internal validation, external validation in a large-scale, multi-center cohort with diverse ethnic and regional backgrounds is an essential prerequisite for its clinical translation—this will be the focus of our future research.

In summary, in this study, we constructed an Nomogram prediction model for recurrent spontaneous abortion based on the expressions of MALAT1, miR-515-5p, and MCL1 mRNA, and verified its clinical application value. The research results showed that the prediction model exhibits high prediction efficiency and accuracy. Since blood samples were collected from non-pregnant women during pre-pregnancy examination, the model is primarily intended for pre-pregnancy risk stratification of women with RSA history-it can help clinicians identify high-risk individuals early and formulate personalized pre-pregnancy intervention strategies (e.g., progesterone supplementation), rather than for diagnosis during pregnancy. Which can provide an intuitive and effective prediction means for clinical doctors to assist them to make more appropriate clinical decisions, thereby reducing the risk of recurrent abortion to the maximum extent and improving the pregnancy success rate. In the future, we need to further expand the sample size, include more risk factors, validate and optimize the model, and in-depth study the risk factors and prediction mechanism in the model, to provide a new scientific basis and clinical guidance for the prevention and treatment of RSA. This study not only provided a new idea and method for the prediction and prevention of RSA, but also provided a new research tool and platform for clinicians and scientific research workers. It should be noted that the Nomogram was constructed based on data from patients aged 18–45 years (in line with the study’s inclusion criteria), and its application scope covers patients aged 18–45 years who meet the inclusion/exclusion criteria (excluding those with genetic diseases, severe organ diseases, etc.), rather than being limited to the average age range of the study population (27–28 years). We believe that in the near future, with the deepening of research and technological progress, RSA prediction and prevention and control will make more significant progress and breakthrough. To further improve the clinical applicability of the constructed Nomogram, we propose optimizing the qPCR workflow and standardizing the application process: First, simplify qPCR detection: Use rapid RNA extraction kits (e.g., spin-column-based kits) to shorten extraction time to 30–40 min, and adopt pre-mixed qPCR reagents to reduce operational errors. Second, standardize the process: Collect only 2 mL fasting peripheral venous blood (instead of 5 mL) to reduce patient discomfort; develop a guide that integrates sample collection, qPCR quality control (RNA purity A260/A280 = 1.8–2.0), and Nomogram risk reading. These measures can help clinicians apply the Nomogram more conveniently and efficiently in clinical practice.

## Data Availability

The raw data supporting the conclusions of this article will be made available by the authors, without undue reservation.

## References

[1] La X Wang W Zhang M Liang L. Definition and multiple factors of recurrent spontaneous abortion. Adv Exp Med Biol. (2021) 1300:231–57. doi: 10.1007/978-981-33-4187-6_11, 33523437

[2] Li L Zhang Z Li H Zhou M Li F Chu C . Research progress on the STAT signaling pathway in pregnancy and pregnancy-associated disorders. Front Immunol. (2024) 14:1331964. doi: 10.3389/fimmu.2023.1331964, 38235138 PMC10792037

[3] Han Y Wang Y Zhang C Li Y Guo J Tian C. Metastasis-associated lung adenocarcinoma transcript 1 induces methyl-CpG-binding domain protein 4 in mice with recurrent spontaneous abortion caused by anti-phospholipid antibody positivity. Placenta. (2023) 137:38–48. doi: 10.1016/j.placenta.2023.04.008, 37068447

[4] Ding J Zhang Y Cai X Yan S Wang J Zhang S . Extracellular vesicles derived from M1 macrophages deliver miR-146a-5p and miR-146b-5p to suppress trophoblast migration and invasion by targeting TRAF6 in recurrent spontaneous abortion. Theranostics. (2021) 11:5813–30. doi: 10.7150/thno.58731, 33897883 PMC8058722

[5] Zhu HL Dai LM Xiong YW Shi XT Liu WB Fu YT . Gestational exposure to environmental cadmium induces placental apoptosis and fetal growth restriction via Parkin-modulated MCL-1 degradation. J Hazard Mater. (2022) 424:127268. doi: 10.1016/j.jhazmat.2021.127268 34583167

[6] Wu L Liu Q Fan C Yi X Cheng B. *MALAT1* recruited the E3 ubiquitin ligase FBXW7 to induce CRY2 ubiquitin-mediated degradation and participated in trophoblast migration and invasion. J Cell Physiol. (2021) 236:2169–77. doi: 10.1002/jcp.30003, 32776544

[7] Fu YY Ren CE Qiao PY Fu Y‐Y Ren C‐E Qiao P‐Y . Uterine natural killer cells and recurrent spontaneous abortion. Am J Reprod Immunol. (2021) 86:e13433. doi: 10.1111/aji.13433 33896061

[8] Li H Cheng L Su S Guo P Wei Z. Pyruvate dehydrogenase kinase 1 regulates the function of human decidual natural killer cells. Am J Reprod Immunol. (2023) 90:e13765. doi: 10.1111/aji.13765, 37766401

[9] Berman JM Shashoua A Olson C Brucker S Thiel JA Bhagavath B. Case series of reproductive outcomes after laparoscopic radiofrequency ablation of symptomatic myomas. J Minim Invasive Gynecol. (2020) 27:639–45. doi: 10.1016/j.jmig.2019.06.009, 31238151

[10] Patki A. Role of dydrogesterone for luteal phase support in assisted reproduction. Reprod Sci. (2024) 31:17–29. doi: 10.1007/s43032-023-01302-z, 37488405

[11] Demirel C Özcan P Tülek F Timur HT Pasin Ö. Initiating luteal phase support with sc progesterone based on low serum progesterone on the transfer day in true natural cycle frozen embryo transfers. Front Endocrinol. (2023) 14:1278042. doi: 10.3389/fendo.2023.1278042, 37937053 PMC10627190

[12] Di X Hao Y Duan Z Ma Y Cao Y Tan Z . Activation of SGK1/ENaC signaling pathway improves the level of decidualization in unexplained recurrent spontaneous abortion. Reprod Sci. (2023) 30:3273–84. doi: 10.1007/s43032-023-01273-1, 37280474 PMC10643273

[13] Luo M Xiao H Wang L Zhao J Gao J Ma W. The expression and clinical significance of three lncRNAs in patients with a missed abortion[J]. Exp Ther Med. (2021) 21:8. doi: 10.3892/etm.2020.9440 33235617 PMC7678617

[14] Wang X Hu H Yu X Liang C Han Y Chen H . Zishen yutai pills promote angiogenesis at the maternal-fetal interface in recurrent spontaneous abortion mice by regulating miR-187/VEGF axis. Drug Des Devel Ther. (2024) 18:407–23. doi: 10.2147/DDDT.S436718 PMC10871043 38370565

[15] Mirinejad S Salimi S Sargazi S Heidari Nia M Sheervalilou R Majidpour M . Association of genetic polymorphisms in long noncoding RNA HOTTIP with risk of idiopathic recurrent spontaneous abortion. Biochem Genet. (2024) 62:2884–906. doi: 10.1007/s10528-023-10571-x, 38038774

[16] Lan M Li H Bao L Li M Lye S Dong X. In vivo evidence of the androgen receptor in association with myometrial cell proliferation and apoptosis. Reprod Sci. (2016) 23:264–71. doi: 10.1177/1933719115602771, 26342051

[17] Park SJ Min JY Kang JS Yang BG Hwang SY Han SH. Chromosomal abnormalities of 19,000 couples with recurrent spontaneous abortions: a multicenter study[J]. Fertil Steril. (2022) 117:1015–25. doi: 10.1016/j.fertnstert.2022.01.011 35216835

[18] Huang X Liu L Xu C . Tissue-resident CD8+ T memory cells with unique properties are present in human decidua during early pregnancy. Am J Reprod Immunol. (2020) 84:e13254. doi: 10.1111/aji.13254 32329123

[19] Zeng L Yang K Liu L Zhang T Liu H Tan Z . Systematic biological and proteomics strategies to explore the regulation mechanism of Shoutai wan on recurrent spontaneous abortion's biological network. J Ethnopharmacol. (2020) 263:113156. doi: 10.1016/j.jep.2020.113156, 32763414

