# Supplementary material for: Construction and validation of nomogram prediction model for recurrent spontaneous abortion based on the expression of MALAT1, miR-515-5p, and MCL1 mRNA
Source: Front Med (Lausanne). 2025 Dec 17;12:1558818. doi: 10.3389/fmed.2025.1558818 (PMC12753443; doi:10.3389/fmed.2025.1558818)
Supplement: Supplementary file 3 [file Table_1.docx]

Supplementary Table S1 Information of Primers Used for Real-time Fluorescent Quantitative PCR

| Primer Name | Gene Symbol | Primer Sequence (5′→3′) | Internal Reference Gene | Product Length (bp) |
| --- | --- | --- | --- | --- |
| MALAT1 primer | MALAT1 | Forward: CCTAACGACTAGCATTGGCA; Reverse: GCACTCTTTCCTGGGCTATC | GAPDH | 168 |
| miR-515-5p primer | miR-515-5p | Forward: UAAUGCUUAAUUGUGAUUGU; Reverse: GTGCAGGGTCCGAGGT | U6 | 72 |
| MCL1 mRNA primer | MCL1 mRNA | Forward: CGGAGATTTGAAGGAGTTTG; Reverse: TCCCGGTTGGTGTAATCTTC | GAPDH | 134 |
| GAPDH primer | GAPDH | Forward: AGGAGTAAGAAACCCTGGAC; Reverse: CTGGGATGGAATTGTGAG | - | 109 |
| U6 primer | U6 | Forward: CTCGCTTCGGCAGCACA; Reverse: AACGCTTCACGAATTTGCGT | - | 97 |
